# RBM47 functions as an anti-oncogene by regulating expression and alternative splicing of cell proliferation and apoptosis associated genes in colorectal cancer cells

**DOI:** 10.1038/s41598-025-05151-5

**Published:** 2025-07-22

**Authors:** Guangshi Liu, Tao Li, Peng Li, Suyan Wei, Kamuran Abulizi

**Affiliations:** 1 People’s Hospital of Xinjiang Uygur Autonomous Region, No. 91 Tianchi Road, Tianshan District, Urumqi, 830011 Xinjiang China; 2 Xinjiang Medical University, No. 91 Tianchi Road, Tianshan District, Urumqi, 830011 Xinjiang China

**Keywords:** RBM47, CRC, Alternative splicing, RNA-seq, Anti-tumor, Cancer genetics, Tumour-suppressor proteins

## Abstract

Transcriptional and post-transcriptional regulation mediated by RNA binding proteins (RBPs) have essential influence on the progression of colorectal cancer (CRC), while the underlying mechanisms need to be clarified. In this study, we focused on RBP-RBM47 and overexpressed its expression in HCT116 cells to evaluate its influence on cellular phenotypes and performed transcriptome sequencing (RNA-seq) to identify the downstream targets. After RBM47 overexpression (RBM47-OE), we found the proliferation level was decreased while apoptosis level was increased. Meanwhile, the invasion and migration ability of HCT116 was also significantly inhibited by RBM47-OE. We identified 216 up and 97 down differentially expressed genes (DEGs) by RBM47-OE, and found they were significantly enriched in immune response, apoptosis, TNF signaling, and autophagy pathways. RBM47-OE can also regulate the splicing pattern of 2541 alternative splicing (AS) events. The regulated AS genes were enriched in cell cycle, DNA damage and repair, mRNA splicing, and cell division associated pathways. To validate the regulation on gene expression and AS, we selected several DEGs and AS events to perform RT-qPCR experiment. The results showed that the expression level of *CASP3*, *CCN1*, and *ATF5*, and the splicing pattern of *CD44* and *MDM2*, were significantly regulated by RBM47-OE in HCT116 cells. In summary, our results demonstrated the anti-tumor function and the globally downstream targets of RBM47 in CRC cells. We extend the cellular and molecular cognition of RBM47 in tumor. The identified molecular targets can also be served as potential targets for CRC treatment in future.

**Supplementary Information:**

The online version contains supplementary material available at 10.1038/s41598-025-05151-5.

## Introduction

Colorectal cancer (CRC) has emerged as one of the most malignant tumors that seriously affect the life quality and survival time of patients, ranking the third and second for morbidity and mortality of all cancer types, respectively^1^. At present, the treatment of colorectal cancer is mainly a comprehensive treatment consisting of surgical procedures and traditional radiotherapy and chemotherapy. However, it has many adverse reactions, long recovery time, and certain limitations. In addition, among newly diagnosed colorectal cancer patients, 20% of them have already metastasized^2,3^. Recently studies demonstrated the important roles of post-transcriptional (PT) regulation in the progression of cancers^4^. As the key regulators of PT regulation, RNA binding proteins (RBPs) coordinately regulate the biogenesis, progression, translation, and degradation of RNAs, thus participating in the development of multiple kinds of cancers^5^including CRC^6,7^. For example, ZMAT3, an RBP induced by p53, binds to the precursor RNA and represses oncogenic isoform of CD44 to inhibit growth of CRC cells^8^. Two RNA-binding motif proteins, RBM24 and RBM38, can bind to the 3’ untranslated region of PTEN to stabilize its expression, thus inhibiting tumorigenesis and progression of CRC, respectively^9,10^. Meanwhile, the functions and molecular mechanisms of most RBPs are largely unknown in CRC.

Recently, we focused on another RBP, RBM47, in CRC. One recent study has summarized the functions of RBM47 in vertebrate development and cancers by post-transcriptional regulation^11^. Meanwhile, RBM47 can also drive the cell fate decisions by regulating p53-p21 axis at transcriptional level^12^ indicating its multifaceted regulatory functions. In cancers, RBM47 has been recognized as a tumor suppressor in multiple tumor types, including breast cancer^13^ non-small cell lung carcinoma^14^ papillary thyroid carcinoma^15^ and hepatocellular carcinoma^16^. It is also reported that RBM47 can promote tumorigenesis of nasopharyngeal carcinoma through both transcriptional and post-transcriptional regulations by cooperating with hnRNPM^17^. In CRC, RBM47 knockdown has also been reported to promote cell migration, invasion and metastases formation in cancer cell lines^18^. RBM47 can also regulate intestinal injury and tumorigenesis of CRC by modifying proliferation, inflammation, and tumorigenic pathways^19^. In summary, these results demonstrate the important roles of RBM47 in inhibiting cancer progression, while the underlying mechanism are not well investigated.

In this study, to better understand how RBM47 regulates the biological processes of CRC cells, we enhanced the expression of RBM47 by transfecting the exogenous RBM47 plasmid in HCT116 cells and performing following experiments to decipher the function and regulatory mechanisms of RBM47. Cellular proliferation and apoptosis experiments were conducted to assess the influence of RBM47 on cell growth, and migration and invasion experiments showed the impact of RBM47 on cell metastasis. More importantly, we performed RNA-seq analysis to illustrate the underlying mechanism and downstream targets of RBM47 in HCT116 cells, including the differentially expressed genes and alternative splicing events. In summary, our study highlights the anti-cancer function of RBM47 in HCT116 cells, as well as the potential molecular targets, especially for the alternative splicing events and genes, regulated by RBM47. Our results extend the understanding of RBM47 function in cancer cells, and the identified molecular targets can be served as potential therapeutic targets for CRC in future.

## Materials and methods

### Cell culture and transfections

We selected the widely used human colorectal cancer HCT116 cell line as research object. The HCT116 cells (Procell, Wuhan, China) were validated by the short tandem repeat (STR) method, and cultured at 37℃ with 5% CO_2_ in McCoy’s 5 A with 10% fetal bovine serum (FBS), 100 µg/mL streptomycin, 100 U/mL penicillin. The RBM47 transcript NM_019027 was inserted into pIRES-hrGFP-1a plasmid, which was transfected into HCT116 cells by Lipofectamine 2000 (Invitrogen, Carlsbad, CA, USA) according to the manufacturer’s protocol. The transfected cells were cultured for 48 h for downstream experiments.

For Actinomycin D (ActD) treatment, after 48 h of transfection with RBM47 and control plasmids, the culture medium was removed and replaced with a medium containing 0.1 nM ActD (HY-17559, MCE). After continuing to culture for 48 h, the cells were collected.

### Cell proliferation assay

The cell proliferation assay was performed using the cell counting kit-8 (CCK-8, CK04, Dojindo, Japan) with 0.5*10^5^ cells as input according to the published procedure^20^. Subsequently, the optical density of the cells was measured with a microplate reader (ELX800, Biotek, USA) at an absorbance of 450 nm to calculate the cell proliferation rate.

### Flow cytometric analysis of cell apoptosis

The Annexin V-FITC/PI cell apoptosis detection kit (40302ES60, Yeasen Biotechnology, China) was used for apoptosis experiment with 1.4*10^5^ cells as input. The cells were mixed with 5 µl Annexin V-FITC incubated at room temperature in the dark for 5 min and 10 µl PI reagents incubated for 5 min as described above respectively. Then the samples were subjected to flow cytometry (FACSCanto, BD, USA) analysis to detect cell apoptosis levels.

### Cell migration assay

In vitro migration assays were performed using transwell chambers (3422, Corning, USA). 3*10^5^ HCT116 cells in 0.2 ml serum-free medium were added to the transwell chambers with 8 μm filter, then the chambers inserted in medium with 600ul 10% FBS (10091148, Gibco, China) served as a chemoattractant in the lower chamber, and incubated for 48 h at 37℃ and 5% CO_2_. Cells remaining on the upper membrane surface of the inserts were then removed with a cotton swab, and the total number of cells that migrated into the lower chamber were fixed by 4% paraformaldehyde (P0099, Beyotime, China) for 10 min, then stained with 0.1% crystal violet (C0121, Beyotime, China). The migration cells were observed and counted under inverted microscope (MF52-N, Mshot, China) at 200× magnification.

### Cell invasion assay

In vitro invasion assays were performed using transwell chambers (3422, Corning, USA). The transwell chambers with 8 μm filter and precoated with a thin layer of Matrigel (356234, BD Biosciences, USA), diluted for 1:8 using serum-free medium, 100 µl diluted matrigel in chambers was incubated for 1 h at 37℃ and 5% CO_2_ and removed unsolidified supernatant. 3 × 10^5^ HCT116 cells in 0.2 ml serum-free medium were added to the inserts, then the transwell chambers inserted in medium with 600 µl 10% FBS (10091148, Gibco, China) served as a chemoattractant in the lower chamber, and incubated for 48 h at 37℃ and 5% CO_2_. Cells remaining on the upper membrane surface of the inserts were then removed with a cotton swab, and the total number of cells that invaded into the lower chamber were fixed by 4% paraformaldehyde (P0099, Beyotime, China) for 10 min, then stained with 0.1% crystal violet (C0121, Beyotime, China). The invasion cells were observed and counted under inverted microscope (MF52-N, Mshot, China) at 200× magnification.

### RNA extraction and sequencing

Total RNAs were extracted from HCT116 cells using TRIzol Reagent (Invitrogen, cat. NO 15596026)following the published method^21^. DNA digestion was carried out after RNA extraction by DNaseI. RNA quality was determined by examining A260/A280 with NanodropTM OneCspectrophotometer (Thermo Fisher Scientific Inc). RNA Integrity was confirmed by 1.5% agarose gel electrophoresis. Qualified RNAs were finally quantified by Qubit3.0 with QubitTM RNA Broad Range Assay kit (Life Technologies, Q10210).

We used 2 µg total RNAs for stranded RNA sequencing library preparation using KCTM Stranded mRNA Library Prep Kit for Illumina (Catalog NO. DR08402, Wuhan Seqhealth Co., Ltd. China) following the manufacturer’s instruction. PCR products corresponding to 200–500 bps were enriched, quantified and finally sequenced on Novaseq 6000 sequencer (Illumina) with PE150 model.

### RNA-Seq raw data clean and alignment

Raw reads containing more than 2-N bases were first discarded. Then adaptors and low-quality bases were trimmed from raw sequencing reads using FASTX-Toolkit (Version 0.0.13). The short reads less than 16nt were also dropped. After that, clean reads were aligned to the human GRCh38 genome by HISAT2^22^ allowing 4 mismatches. Uniquely mapped reads were used for gene reads number counting and fragments per kilobase of transcript per million fragments mapped (FPKM) calculation^23^. The statistical power of this experimental design, calculated in RNASeqPower is 1.

### Differentially expressed genes (DEG) analysis

The R Bioconductor package DESeq2^24^ was utilized to screen out the differentially expressed genes (DEGs). The thresholds *P*-value < 0.01 and fold change > 1.5 were set as the cut-off criteria for identifying DEGs.

### Alternative splicing analysis

The alternative splicing events (ASEs) and regulated alternative splicing events (RASEs) between the samples were defined and quantified by using the ABLas pipeline as described previously^25,26^. In brief, ABLas detection of ten types of ASEs was based on the splice junction reads, including exon skipping (ES), alternative 5’ splice site(A5SS), alternative 3’splice site(A3SS), mutually exclusive exons (MXE), mutually exclusive 5’UTRs (5pMXE), mutually exclusive 3’UTRs (3pMXE), cassette exon, A3SS&ES, and A5SS&ES.

To assess RBP regulated ASE, Student’s t-test was performed to evaluate the significance of the ratio alteration of AS events. Those events which were significant at P-value cutoff corresponding to a false discovery rate cutoff of 5% were considered RBP regulated ASEs.

### Functional enrichment analysis

To sort out functional categories of DEGs, Gene Ontology (GO) terms and KEGG pathways were identified using KOBAS 2.0 server^27^. Hyper geometric test and Benjamini-Hochberg FDR controlling procedure were used to define the enrichment of each term.

### Statistical analysis

For statistical analysis, error bars represent mean ± SEM. Unpaired and two-tail Student’s *t*-test was used to analyze the statistical difference between two groups.

## Results

### RBM47 acts as a tumor suppressor in CRC patients and HCT116 cell line

**Fig. 1:**
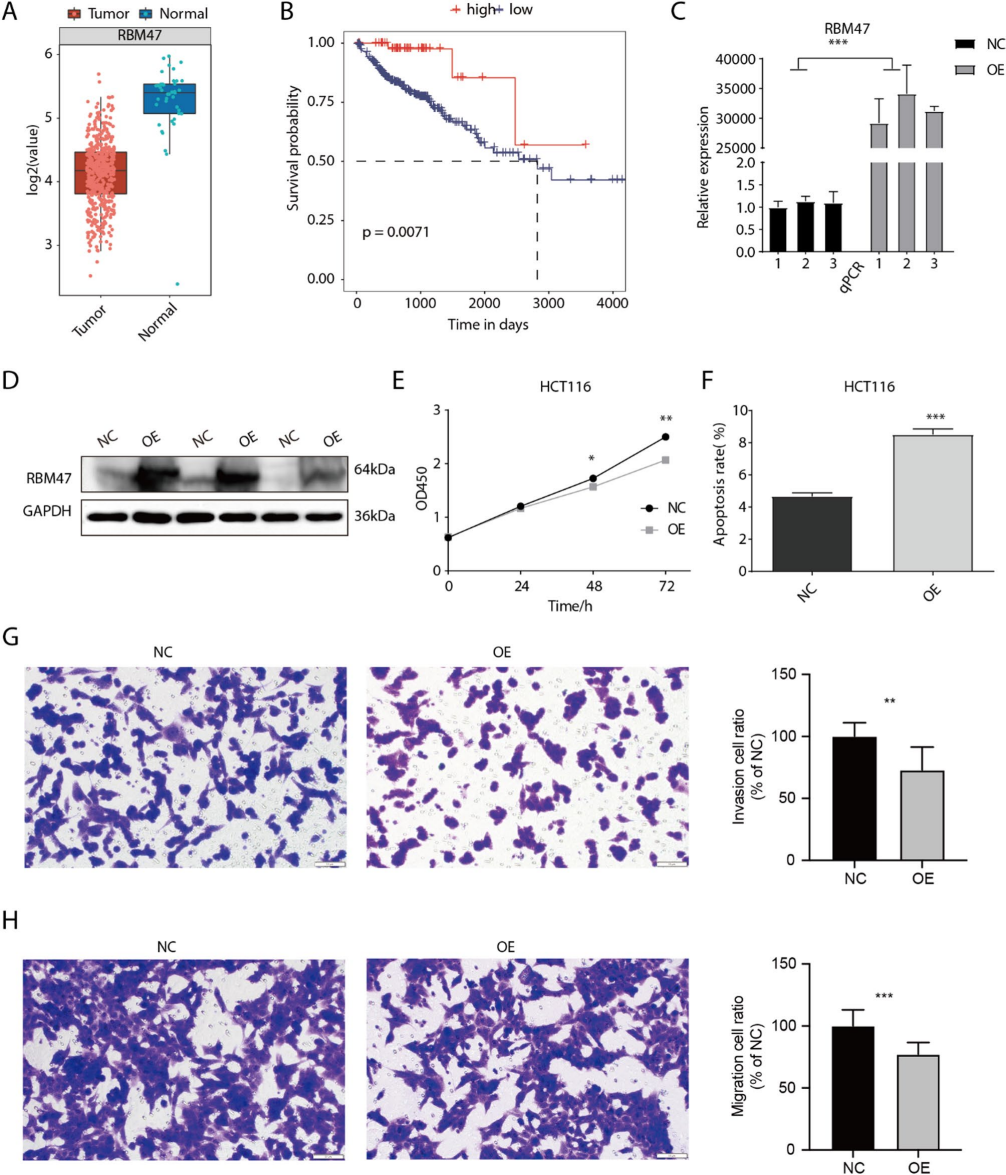
RBM47 functions as a tumor suppressor in colorectal cancer. (A) Box plot showing the expression level of RBM47 in tumor and adjacent normal tissues of COAD patients from TCGA. (B) The survival curve of RBM47 in colorectal cancer in TCGA. (C) The histogram showed the RT-qPCR results of control and treatment samples. *** *P*-value < 0.001. (D) The result of western blot experiment showed that the RBM47 over expression was successful. (E) The proliferation results of HTC116 after RBM47 overexpression. * *P*-value < 0.05; ** *P*-value < 0.01. (F) The apoptosis results of HTC116 after RBM47 overexpression. *** *P*-value < 0.001. (G) The invasion results of HCT116 cells after RBM47 overexpression. ** *P*-value < 0.01. (H) The migration results of HCT116 cells after RBM47 overexpression. *** *P*-value < 0.001.

Previous study has demonstrated that RBM47 knockdown can promote cell migration, invasion and metastases formation of CRC cell lines^18^ while the underlying mechanism is largely unknown. By investigating the cancer genome atlas (TCGA) dataset of colon adenocarcinoma (COAD) patients, we found RBM47 was significantly downregulated in tumor samples compared with adjacent normal samples (Fig. 1A). At the same time, higher expression level of RBM47 in COAD patients was associated with better overall survival time compared with patients with lower RBM47 level (Fig. 1B). To further confirm the cellular functions of RBM47, we performed RBM47 overexpression (RBM47-OE) because of the low expression level of RBM47 in HCT116 cell line. Both RT-qPCR and western blot experiments confirmed the successful overexpression of RBM47 in HCT116 cells (Fig. 1C-D, Figure S1A-B). Then we explored the cellular functions of RBM47-OE by checking its influence on cell proliferation and apoptosis. The results demonstrated that RBM47-OE significantly inhibited the proliferation level of HCT116 cells at 48 and 72 h (Fig. 1E), while RBM47-OE significantly promoted the apoptosis level of HCT116 cells (Fig. 1F, Figure S1C). Then we performed cell invasion and migration experiments, and found that RBM47-OE significantly decreased the abilities of cell invasion (Fig. 1G) and migration (Fig. 1H) of HCT116 cells. These results were complementary to previous conclusion and further confirmed the tumor-suppressing function of RBM47 in CRC cell line.

### RBM47-OE globally modulated the transcriptome profile of HCT116 cells

**Fig. 2:**
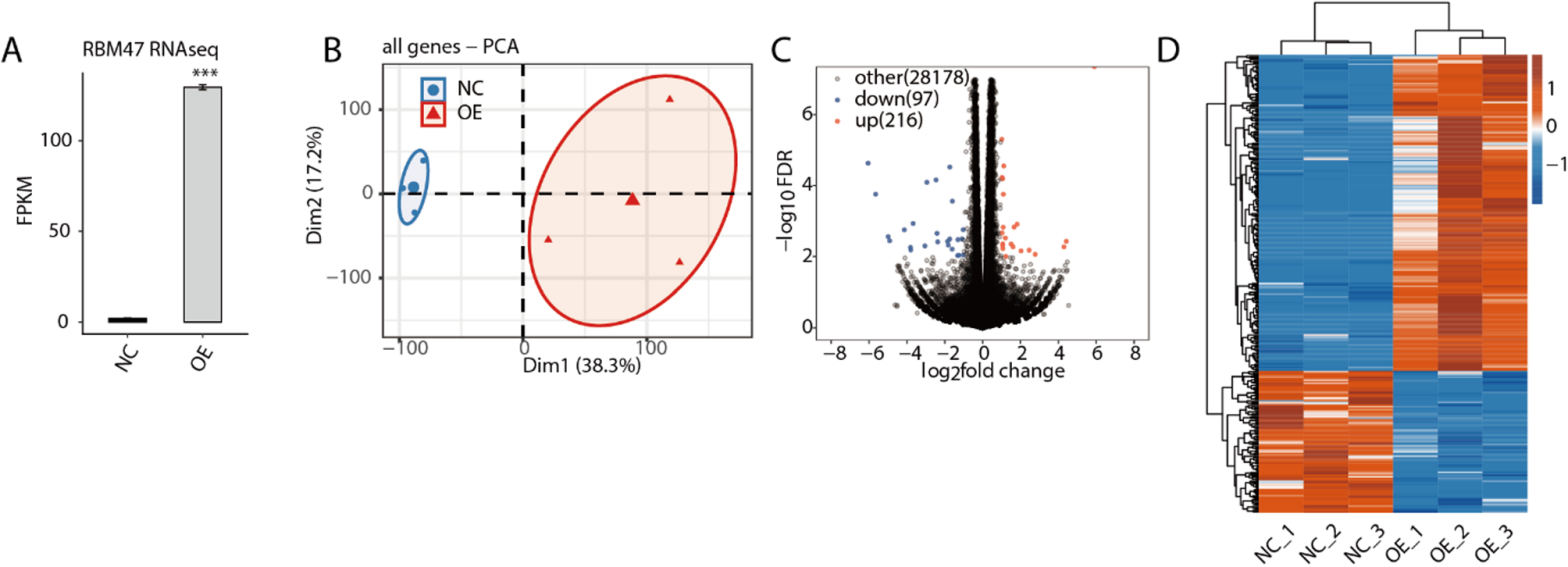
RBM47-OE affect gene expression profiles of HCT116 cells. (A) Bar plot showing the expression pattern and statistical difference of DEGs for RBM47. Error bars represent mean ± SEM. *** *P*-value < 0.001. (B) PCA base on FPKM value of all detected genes RBM47 overexpression. The ellipse for each group is the confidence ellipse. (C) Volcano plot showing all differentially expressed genes (DEGs) between RBM47-OE and Ctrl samples. (D) Hierarchical clustering heat map showing expression levels of all DEGs.

To further explore how RBM47 modulates the cellular phenotypes of HCT116 cells, we performed global RNA-seq analysis and identified the transcriptome difference between RBM47-OE vs. NC samples. After calculating the FPKM values of expressed genes, we found that RBM47 expression was also significantly increased in OE samples (Fig. 2A), confirmed the detection by RT-qPCR. Principal component analysis (PCA) for all detected genes demonstrated the clear separation between RBM47-OE and NC samples at the first component (Fig. 2B), indicating that RBM47 can globally modulate the transcriptome profile of HCT116 cells. Thus, we predicted the differentially expressed genes (DEGs) using fold change > 1.5 and *p*-value < 0.01 as thresholds, and finally obtained 216 up and 97 down DEGs (Fig. 2C). The fact that more up DEGs than down DEGs indicates that RBM47-OE prefers to promote gene expression in HCT116 cells. By plotting the expression pattern of DEGs, we found they showed consistent expression pattern among the three replicates for each group (Fig. 2D).

### DEGs induced by RBM47-OE were enriched in cell cycle and apoptosis pathways

**Fig. 3:**
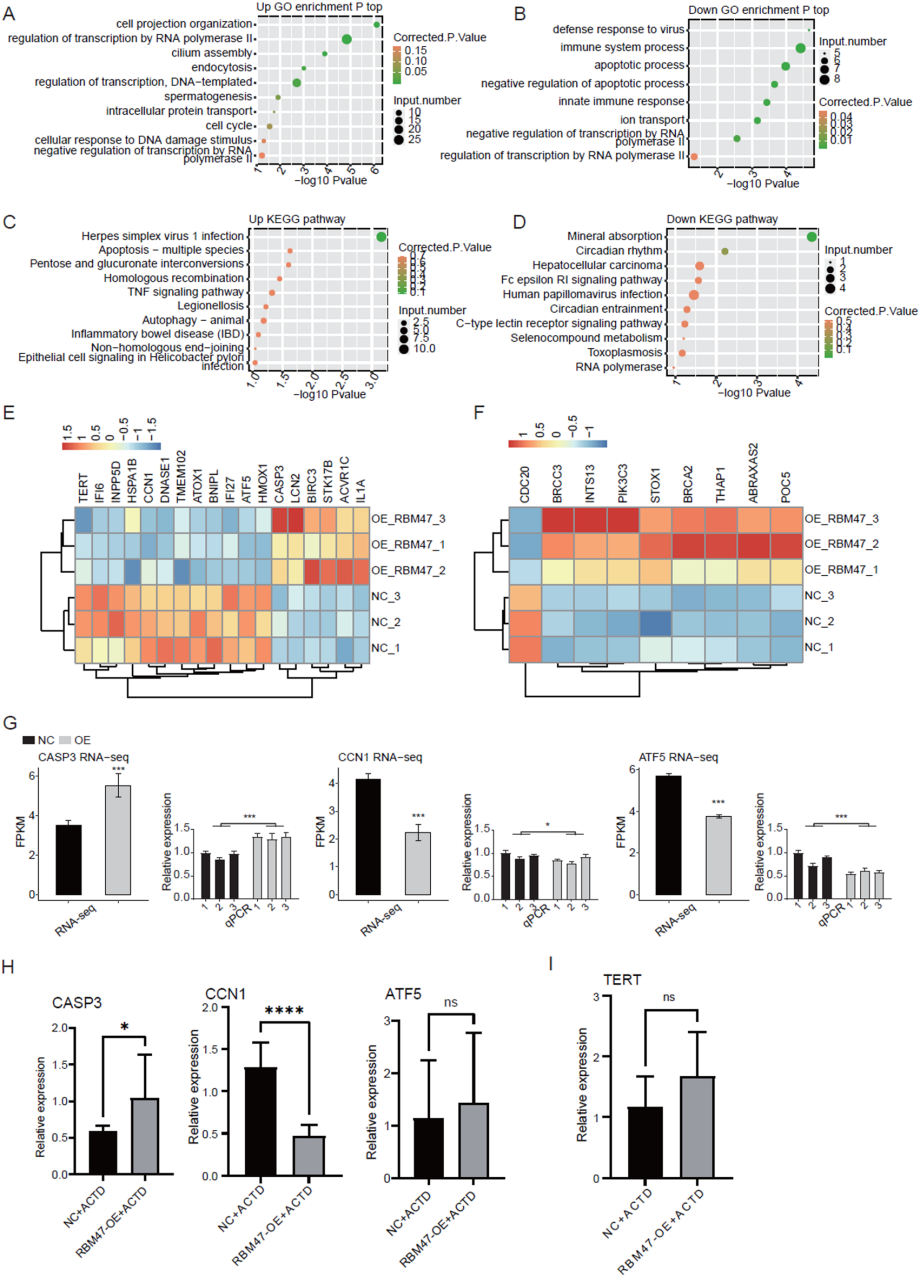
RBM47 broadly regulate expression of cell cycle and apoptosis associated genes in HTC116 cells. (A) The Scatter plot exhibiting the most enriched GO biological process results of the up-regulated DEGs. (B) The Scatter plot exhibiting the most enriched GO biological process results of the down-regulated DEGs. (C) The Scatter plot exhibiting the most enriched KEGG pathway results of the up-regulated DEGs. (D) The Scatter plot exhibiting the most enriched KEGG pathway results of the down-regulated DEGs. (E) Clustering heatmap showing the DEGs involved in apoptosis pathway. (F) Clustering heatmap showing the DEGs involved in cell cycle pathway. (G) Bar plot showing the expression pattern and statistical difference of DEGs for some important genes. (H) Bar plot showing the RT-qPCR results and statistical difference of DEGs in (G) by ActD treatment. (I) The same as (H) but for *TERT* gene. Error bars represent mean ± SEM. **** *P*-value < 0.0001, *** *P*-value < 0.001, ** *P*-value < 0.01, * *P*-value < 0.05.

To investigate the functions of DEGs induced by RBM47-OE, we performed functional enrichment analysis using GO and KEGG databases. We found the up DEGs were enriched in cell projection organization, regulation of transcription by RNA polymerase II, cilium assembly, and endocytosis GO pathways (Fig. 3A), whereas the down DEGs were mainly enriched in defense response to virus, immune system process, apoptotic process, and negative regulation of apoptotic process pathways (Fig. 3B). For KEGG analysis, apoptosis-multiple species, TNF signaling pathway, and autophagy-animal pathways were emerged in the top pathways of up DEGs (Fig. 3C). Several cancer-associated pathways, including hepatocellular carcinoma and human papillomavirus infection were detected in down DEGs (Fig. 3D). Several apoptosis-associated genes, including CASP3, ACVR1C, LCN2, BIRC3, IL1A, and STK17B, were significantly upregulated by RBM47-OE, whereas several other apoptotic genes, including *HSPA1B*,* IFI6*,* HMOX1*,* TMEM102*,* ATOX1*,* ATF5*,* CCN1*,* DNASE1*,* TERT*,* IFI27*,* BNIPL*, and *INPP5D*, were downregulated by RBM47-OE (Fig. 3E). For cell cycle genes, *INTS13*,* ABRAXAS2*,* BRCC3*,* POC5*,* THAP1*,* PIK3C3*,* STOX1*, and *BRCA2* were upregulated, whereas *CDC20* was downregulated (Fig. 3F). To confirm the regulation of RBM47 on DEGs, we selected three DEGs and performed RT-qPCR experiment. *CASP3* was upregulated, and *CCN1* and *ATF5* were downregulated after RBM47-OE by RT-qPCR (Fig. 3G). In addition, we also used Actinomycin D (actD) to inhibit the transcription process of HCT116 cells. Further RT-qPCR experiment demonstrated that RBM47-OE enhanced *CASP3* expression, inhibited *CNN1* expression, but not significantly changed the expression of *ATF5* under ActD treatment (Fig. 3H). Meanwhile, we also tested the expression pattern of TERT and did not find significant difference between NC and RBM47-OE under ActD treatment (Fig. 3I). In summary, these results indicated that RBM47 can regulate gene expression at both transcriptional and post-transcriptional levels and were consistent with the discovery that RBM47-OE significantly changed the proliferation and apoptosis levels of HCT116 cells.

### RBM47-OE had profound impact on the alternative splicing pattern of HCT116 cells

**Fig. 4:**
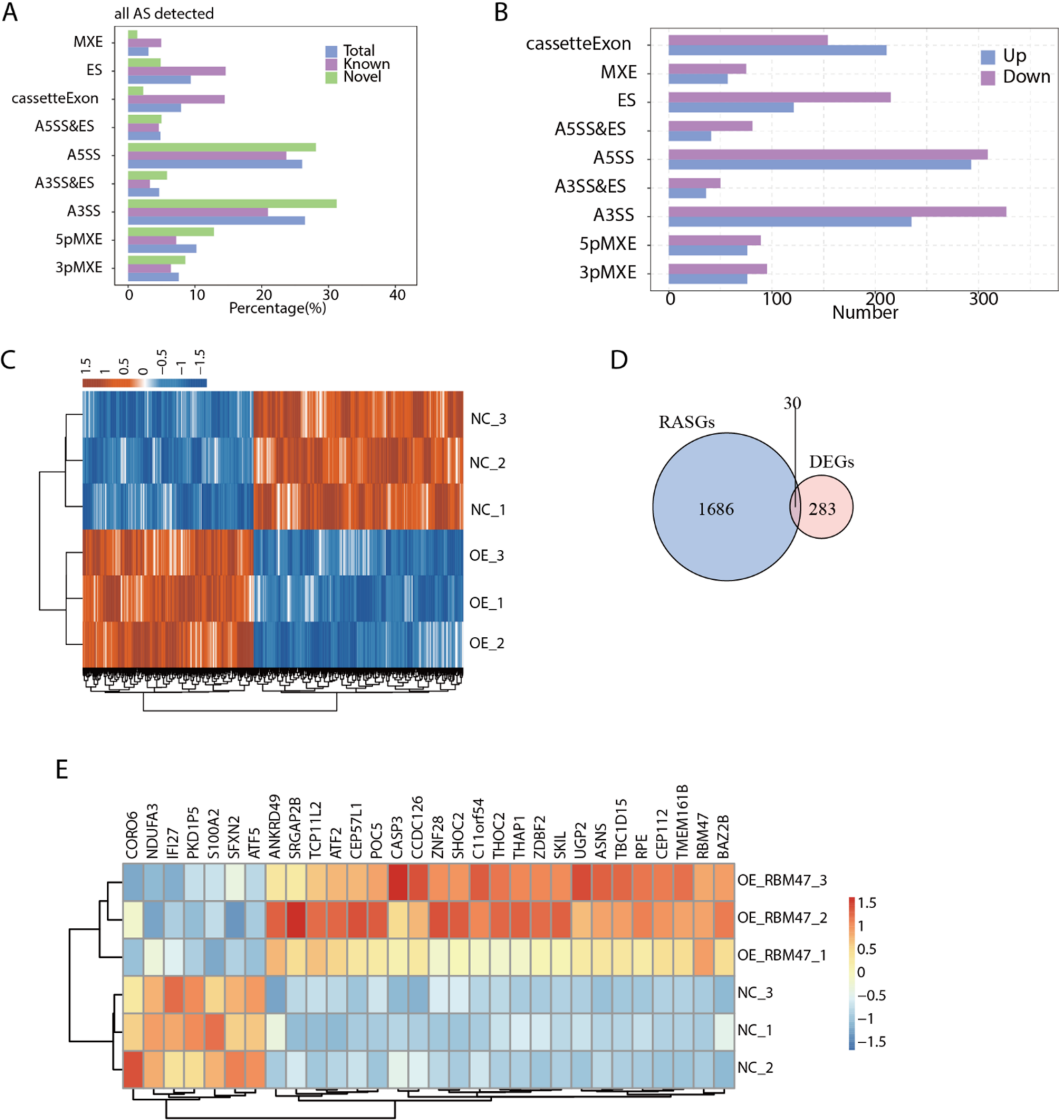
RBM47-OE broadly change the alternative splicing patterns in HCT116 cells. (A) Bar plot showing the RBM47 all regulated alternative splicing event. (B) Bar plot showing the RBM47 regulated alternative splicing events (RASEs). (C) Hierarchical clustering heat map showing expression levels of RBM47 regulated alternative splicing event (RASE). (D) Venn diagram showing the overlapped genes between RASGs and DEGs. (E) Hierarchical clustering heat map showing expression levels of the overlapped 30 genes in (D).

RBM47 has been reported to regulate alternative splicing of cancer-related genes in nasopharyngeal carcinoma^17^. Thus, we dedicated to explore the AS profile alteration by RBM47-OE in HCT116 cells. We universally detected the known and novel AS events (ASEs) from the aligning result, and found that A3SS and A5SS were the two most detected ASEs (Fig. 4A). To further illustrate how RBM47-OE regulated AS profile, we predicted the regulated ASEs (RASEs) by RBM47, and finally detected 2541 RASEs. The results showed that A5SS and A3SS were also the top two RASE types if we divided cassette exon and exon skipping (ES) into two types (Fig. 4B). Meanwhile, we found more exons were included than excluded by RBM47-OE as we detected more excluded (down) ES and more included (up) cassette exon events (Fig. 4B), indicating that RBM47-OE had the ability to include exons during splicing progression. By plotting the ratios of these RASEs, we found these RASEs were consistently regulated in all the three replicates (Fig. 4C). Finally, we performed an overlap analysis for RASE genes (RASGs) and DEGs, and detected 30 overlapped genes (Fig. 4D). The much more RASG number indicates that RBM47 probably has more important roles in AS regulation that gene expression modulation in HCT116 cells.

### RBM47 broadly regulates the AS profile of genes involved in cell cycle and DNA repair

**Fig. 5:**
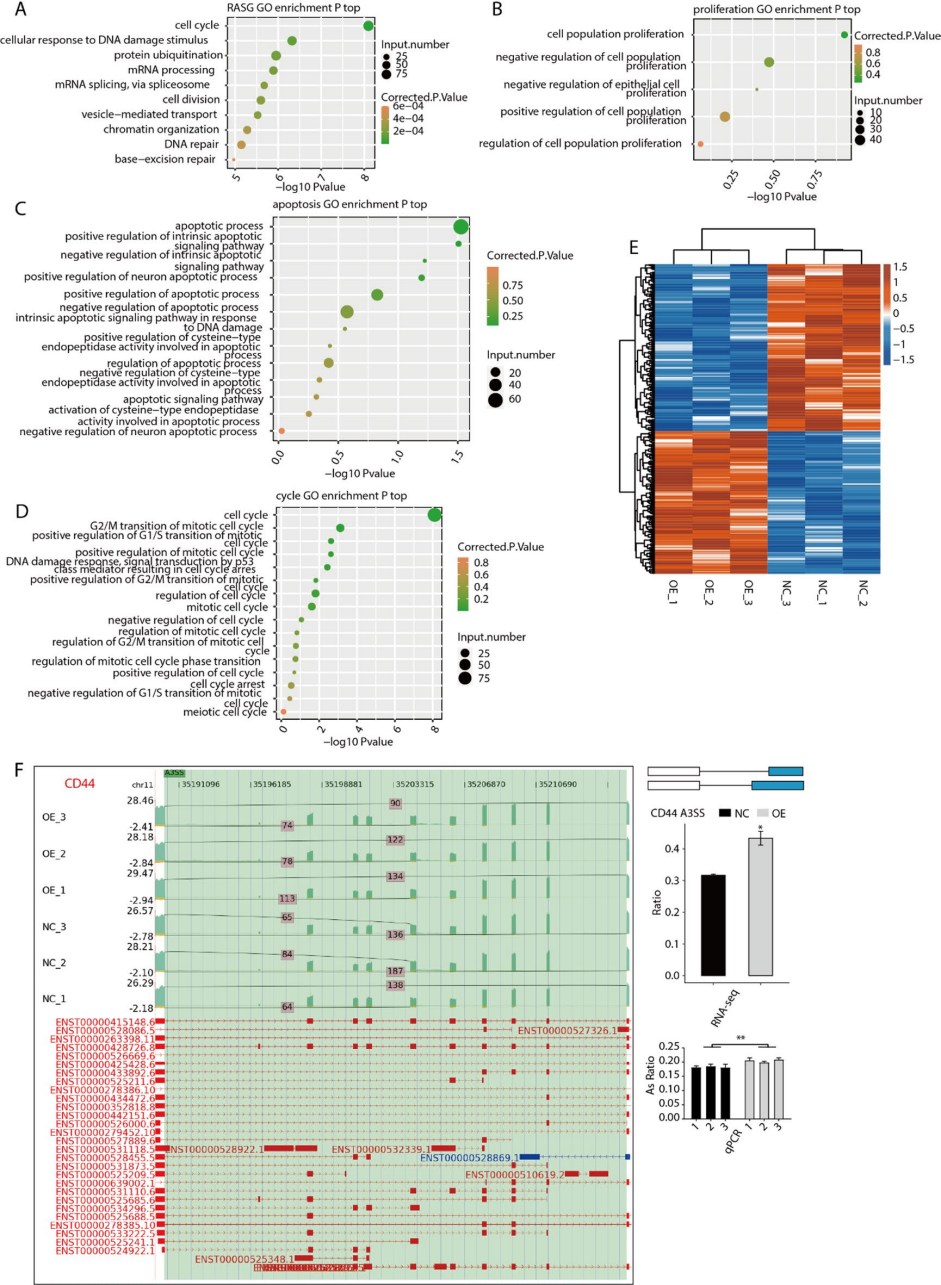
RBM47 regulates alternative splicing of cell cycle, proliferation and apoptosis associated genes in HCT116 cells. (A) Scatter plot exhibiting the most enriched GO biological process results of the RASGs. (B) Scatter plot exhibiting all the proliferative pathways GO biological process results of the RASGs. (C) Scatter plot exhibiting all the apoptotic pathways GO biological process results of the RASGs. (D) Scatter plot exhibiting all the cycle pathways GO biological process results of the RASGs. (E) Hierarchical clustering heat map showing expression levels of all associated genes about (B), (C) and (D) in RBM47 regulated alternative splicing events (RASEs). (F) RBM47 regulates alternative splicing of *CD44*. Left panel: IGV-sashimi plot showing the regulated alternative splicing events and binding sites across mRNA. Reads distribution of RASE is plotted in the up panel and the transcripts of each gene are shown below. Right panel: The schematic diagrams depict the structures of ASEs. RNA-seq validation of RASEs was shown at the bottom of the right panel. Error bars represent mean ± SEM. *** *P*-value < 0.001, ** *P*-value < 0.01, * *P*-value < 0.05.

AS RBM47-OE significantly modulated the AS pattern of 1716 RASGs (Fig. 4D), then we explored the enriched functions of these RASGs. We found these RASGs were dominantly enriched in cell cycle, cellular response to DNA damage stimulus, protein ubiquitination, mRNA processing, mRNA splicing via spliceosome, cell division, vesicle-mediated transport, chromatin organization, DNA repair, and base-excision repair GO BP pathways (Fig. 5A). KEGG enrichment analysis for these RASGs showed they were enriched in shigellosis, autophagy, and ubiquitin mediated proteolysis pathways (Figure S2A). Among the detected GO BP pathways, we found there were five pathways belonging to cell proliferation, thirteen pathways belonging to apoptosis, and sixteen pathways belonging to cell cycle (Fig. 5B-D, Figure S2B), although a lot of them were not significant. These results indicates that RASGs contained genes involved in cell proliferation, apoptosis, and cell cycle, which may have essential regulation on the cellular phenotypes modulated by RBM47. By clustering the RASE ratios of RASGs from proliferation, apoptosis, and cell cycle, we found these RASEs showed consistent dysregulation in three replicates by RBM47-OE (Fig. 5E). Meanwhile, we also found one RASE from CD44, an important factor in tumor metastasis^28^ was significantly dysregulated by RBM47-OE, and also confirmed by RT-qPCR experiment (Fig. 5F). MDM2, an E3 ubiquitin ligase protein that is a candidate target for tumor therapy^29^ also showed AS dysregulation with an A5SS event, and was confirmed by RT-qPCR (Figure S2C). Taken together, these results demonstrated that RBM47 can globally regulate the AS profile that are associated with cell cycle, DNA damage and repair, and mRNA splicing.

## Discussion

The previous research suggests that RBM47 has an anti-proliferation and anti-metastasis effect in several cancer cells^30–32^. Our analysis of the TCGA database also confirmed that the expression of RBM47 was closely associated with the progression of CRC. Additionally, when we overexpressed RBM47 in the HCT116 cell line, we observed the inhibited cell proliferation and promoted apoptosis. These findings align with previous studies indicating that reducing RBM47 expression enhances CRC cell lines’ migration, invasion, and metastasis^19^. Moreover, we next performed RNA-seq analysis to decipher the underlying mechanisms and molecular targets about RBM47’s anti-cancer function in HCT116 cells. In summary, our results highlight the important roles in cellular phenotype and transcriptome modulation of RBM47, which extends our understanding about the regulatory mechanisms of RBM47 in CRC.

Through RNA-seq analysis, we found that RBM47-OE globally regulated the transcriptomic landscape of HCT116 cells. We identified 313 DEGs and 1716 RASEs. Functional enrichment analysis revealed that these DEGs and RASEs were primarily enriched in biological processes such as cell cycle, apoptosis, and DNA repair, which are closely related to the tumor-suppressive role of RBM47. RBM47 can act as an interferon-stimulating gene to enhance the antiviral effect of the host by stabilizing the mRNA of the target gene, revealing its role in antiviral immunity. The ISGylation of RBM47 can regulate immune activation, which is a well-established antiviral mechanism, and participate in DNA repair, autophagy, transcription, protein translation, and other cellular processes^33,34^. Interestingly, the DEGs of RBM47-OE are enriched in these related pathways, which implies RBM47 may regulate CRC cell proliferation by itself ISGylation, stabilizing mRNA, and alternative splicing of downstream immune response and tumor suppression-related genes.

We specifically focused on the effect of RBM47 on cell cycle and apoptosis-related genes. For instance, apoptosis-related gene such as CASP3, was significantly upregulated after RBM47-OE, while genes like CCN1 and ATF5 were downregulated. Cell cycle genes such as INTS13 and BRCA2 were also positively regulated by RBM47-OE. CCN1 is a cysteine-rich intercellular matrix protein, high expression of CCN1 is associated with the progression and poor prognosis of multiple cancers and is involved in tumor growth, angiogenesis, and cancer cell adhesion, migration, and invasion^35–38^. ATF5 modulates many genes’ expression and regulates the cell cycle, unfolded protein response (UPR), anti-apoptosis, immune, and other signaling pathways, playing a crucial role in the survival and proliferation of cancer cells. Its expression is negatively correlated with the survival rate of patients with various types of cancer, making it a popular target for cancer therapy^39–44^. CASP3 plays a key role in apoptosis and is a biomarker of apoptosis. Activation of the CASP3/GSDME signaling pathway can induce pyroptosis or apoptosis, which in turn affects the growth of tumors and the survival rate of cancer patients^45–47^. The expression of *CASP3* is up-regulated in most cancers, but down-regulated in colon adenocarcinoma and rectum adenocarcinoma, indicating that CASP3 has different regulatory functions in various tumors^48^.

What’s more, we observed a broad effect of RBM47 on alternative splicing patterns, which may further regulate the functions of these genes. Cell cycle, DNA damage and repair, mRNA splicing, and cell division were the top enriched pathways for RASGs (Fig. 5A). It has been reported that RBM47 regulates the exon inclusion of TJP1 and inhibits expression of TJP1-α isoform, which in turn enhances the assembly of actin stress fibers and promotes cellular migration in A549 cells^49^. The identified RASGs in this study were tightly associated with the cellular functions of RBM47 in HCT116 cells. We propose that RBM47 can broadly alter the splicing pattern of these genes and modifies the expression level, amino acid sequences or structures of the encoded proteins, and finally changes their functions in tumor cells. RBM47 modulates the splicing of CD44, which is a marker of cancer stem cells. It is reported that CD44 has tumor-processing function and promotes EMT and metastasis of colon cancer cells^28,50^. RBM47 also altered the splicing of MDM2 (Figure S2C). Several studies have demonstrated that MDM2 is a new therapeutic option in colon cancer treatment^51^ as both the inhibitor and anti-sense oligonucleotide of MDM2 have anti-tumor activity by inhibiting growth and proliferation of colon cancer cells^52,53^. These results indicate that RBM47 inhibits growth and metastasis of HCT116 cells probably by regulating the splicing pattern of CD44 and MDM2 transcripts. Finally, the AS results were consistent with our RT-qPCR detection results, further confirming the anti-tumor function of RBM47 in CRC. Further studies are necessary to decipher the functions of the alternative spliced forms of CD44 or MDM2, which are not deeply investigated in this study.

Although our study provides insights into the potential mechanisms of RBM47’s role in CRC, it has some limitations. First, we conducted experiments using only one cell line, which may limit the generalizability of the results. In addition, we did not perform RBM47 knockdown experiments in cell and animal model to validate our findings. Furthermore, as an RNA-binding protein, it is worth exploring whether the regulatory effects of RBM47 may differ between left-sided and right-sided colorectal cancer due to the different primary sites^54–56^. Future studies should include multiple CRC cell lines and in vivo animal models to explore the function and molecular mechanism of RBM47.

## Conclusions

In conclusion, our findings highlight the significance of RBM47 as a tumor suppressor gene in CRC. RBM47 plays a role in cell proliferation and apoptosis via regulating gene expression and alternative splicing, providing potential molecular targets and valuable information for the development of novel CRC treatment strategies.

## Electronic supplementary material

Below is the link to the electronic supplementary material.

Supplementary Material 1

Supplementary Material 2

## Data Availability

The raw RNA-seq data has been deposited in the NCBI GEO database with accession ID GSE282110. Editors and reviewers can contact the corresponding author Guangshi Liu for for the data availability request.
