# Supplementary material for: RBM47 functions as an anti-oncogene by regulating expression and alternative splicing of cell proliferation and apoptosis associated genes in colorectal cancer cells
Source: Sci Rep. 2025 Jul 22;15:26685. doi: 10.1038/s41598-025-05151-5 (PMC12284113; doi:10.1038/s41598-025-05151-5)
Supplement: Supplementary file 1 — Supplementary Material 1 [file 41598_2025_5151_MOESM1_ESM.pdf]

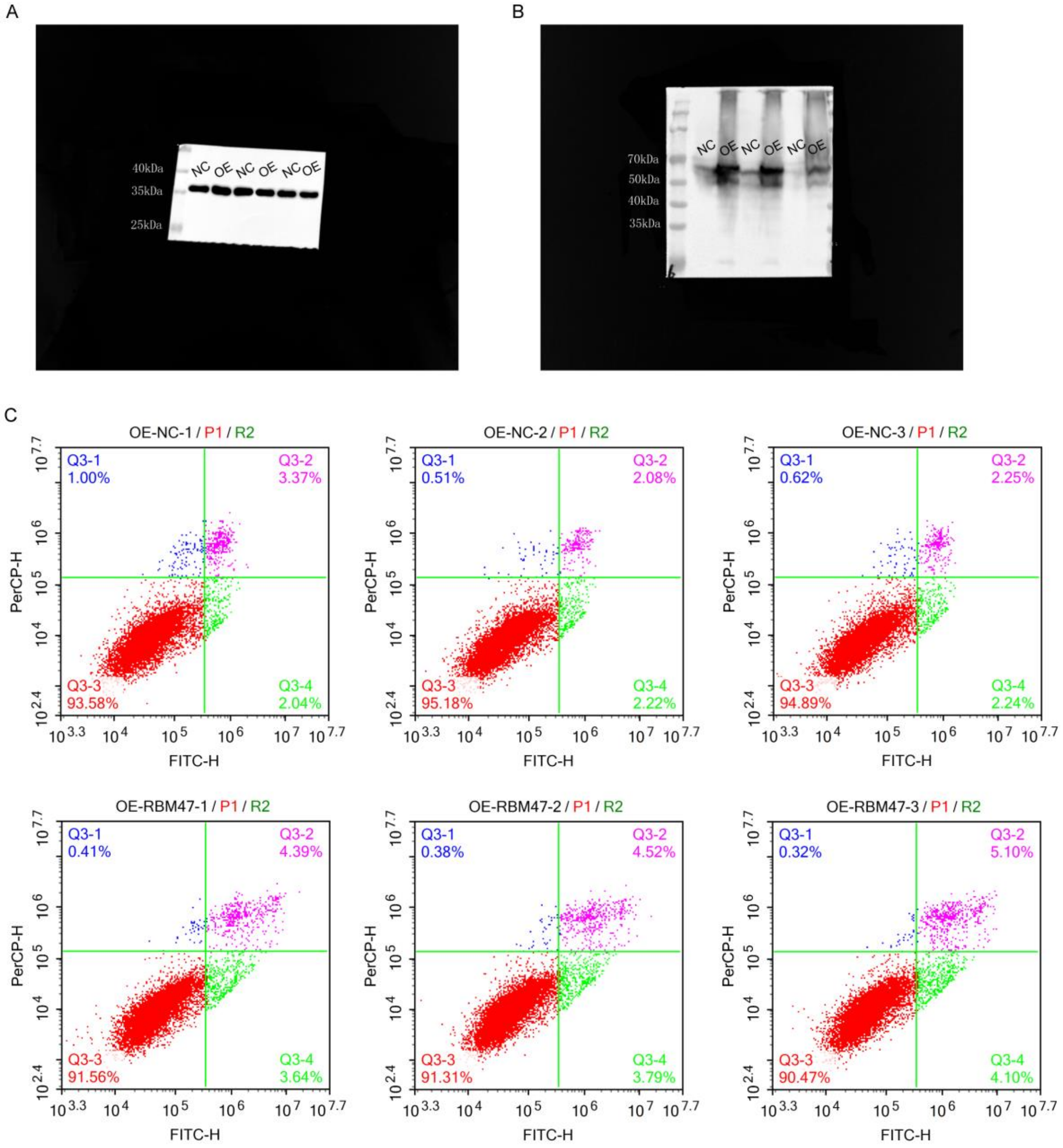

Figure S1. Results of western blot experiment and flow cytometry

(A) The result of western blot experiment of GAPDH. (B) The result of western blot experiment of RBM47. (C) Flow cytometry results.

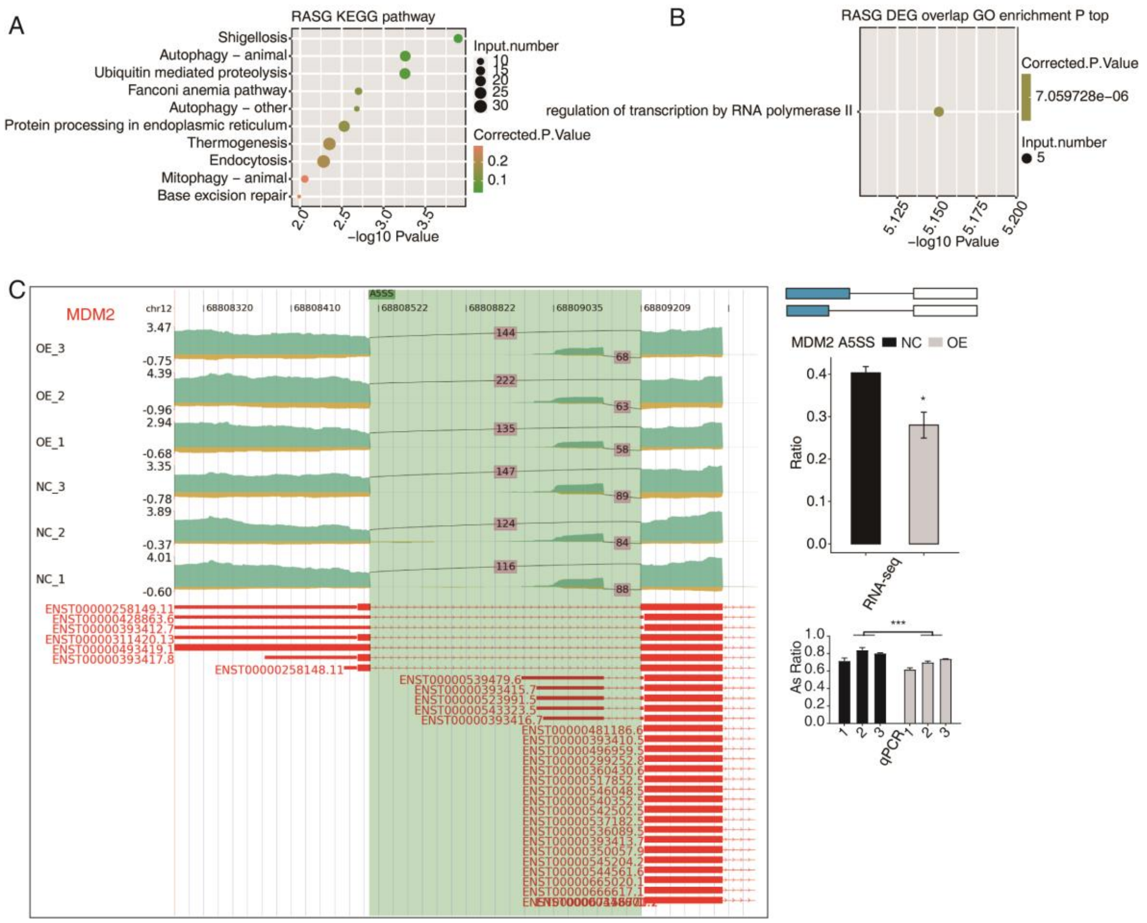

Figure S2. RBM47 regulates alternative splicing in HCT116 cells.

(A) Scatter plot exhibiting the most enriched KEGG pathway results of the regulated alternative splicing genes (RASEs). (B) Scatter plot exhibiting the most enriched GO biological process results of the overlapped genes between RASGs and DEGs. (C) RBM47 regulates alternative splicing of ABI1. Left panel: IGV-sashimi plot showing the regulated alternative splicing events and binding sites across mRNA. Reads distribution of RASE is plotted in the up panel and the transcripts of each gene are shown below. Right panel: The schematic diagrams depict the structures of ASEs. RNA-seq validation of ASEs are shown at the bottom of the right panel. Error bars represent mean ± SEM. ***P-value < 0.001, ** P-value < 0.01, * P-value < 0.05.
