# Supplementary material for: RBM47 functions as an anti-oncogene by regulating expression and alternative splicing of cell proliferation and apoptosis associated genes in colorectal cancer cells
Source: Sci Rep. 2025 Jul 22;15:26685. doi: 10.1038/s41598-025-05151-5 (PMC12284113; doi:10.1038/s41598-025-05151-5)
Supplement: Supplementary file 2 — Supplementary Material 2 [file 41598_2025_5151_MOESM2_ESM.docx]

**Table S1. The primer sequence table for RT-qPCR experiment.**

| CCN1-F | AGCCTCGCATCCTATACA |
| --- | --- |
| CCN1-R | GTCATTGGTAACTCGTGTG |
| LCN2-F | GGAAGTGGTATGTGGTAGG |
| LCN2-R | GACGGAGGTGACATTGTAG |
| CASP3-F | GTTAGACTTCTACAACGATCC |
| CASP3-R | TTGTTTGTGTGCTTCTGAG |
| ATF5-F | TCTTCCACTTTCGCCTTG |
| ATF5-R | AGGAGTGACATGGCTGTA |
| ATOX1-F | CCTTTGGTCCATCCTGTG |
| ATOX1-R | GGTCTGCATTGAATCTGAG |
| HSPA1B-F | TCATCAGCGGACTGTACC |
| HSPA1B-R | CCTAATCCACCTCCTCAATG |
| TERT-F | GCCGATTGTGAACATGGACTACG |
| TERT-R | GCTCGTAGTTGAGCACGCTGAA |
| ABI1-M-F | TGAGCCCGGGGCTGGTCCAATA |
| ABI1-AS-F | AGCCCGGGGCTGCTGGTCCAATA |
| ABI1-M/AS-R | GGAAGTAGTGGAGGAAGTG |
| CD44-M/AS-F1 | GCACTTCAGGAGGTTACAT |
| CD44-AS-R1 | CTCATCAAAGTGGTAGCAGG |
| CD44-M-R1 | GTGGTTGAAATGGTAGCAGG |
| CD44-M/AS-F2 | GCACTTCAGGAGGTTACAT |
| CD44-AS-R2 | GTGGTTGAAATGGTAGCAGG |
| CD44-M-R2 | TCTTGGTCTCTGGTAGCAGG |
| CD44-M/AS-F3 | GCACTTCAGGAGGTTACAT |
| CD44-AS-R3 | GTTGCCTGGATGGTAGCAGG |
| CD44-M-R3 | TCTTGGTCTCTGGTAGCAGG |
| MDM2-M-F | TGAGGAGCAGGCAAATGTGC |
| MDM2-AS-F | TGCTGATCCAGGCAAATGTGC |
| MDM2-M/AS-R | CAGGGTCTCTTGTTCCGAAG |
| Internal control |  |
| hum-GAPDH-F | GGTCGGAGTCAACGGATTTG |
| hum-GAPDH-R | GGAAGATGGTGATGGGATTTC |
